# Overexpression of Plg-R_KT_ protects against adipose dysfunction and dysregulation of glucose homeostasis in diet-induced obese mice

**DOI:** 10.1080/21623945.2023.2252729

**Published:** 2023-09-04

**Authors:** Lindsey A. Miles, Hongdong Bai, Sagarika Chakrabarty, Nagyung Baik, Yuqing Zhang, Robert J. Parmer, Fahumiya Samad

**Affiliations:** a Department of Molecular Medicine, Scripps Research, La Jolla, CA, USA; b Department of Medicine, Veterans Administration San Diego Healthcare System, San Diego, CA, USA; c Department of Cell Biology, San Diego Biomedical Research Institute, San Diego, CA, USA; d Department of Medicine, University of California San Diego, La Jolla, CA, USA

**Keywords:** Plg-R_KT_, adipose tissue, adipose inflammation, insulin resistance, brown fat thermogenesis

## Abstract

The plasminogen receptor, Plg-R_KT,_ is a unique cell surface receptor that is broadly expressed in cells and tissues throughout the body. Plg-R_KT_ localizes plasminogen on cell surfaces and promotes its activation to the broad-spectrum serine protease, plasmin. In this study, we show that overexpression of Plg-R_KT_ protects mice from high fat diet (HFD)-induced adipose and metabolic dysfunction. During the first 10 weeks on the HFD, the body weights of mice that overexpressed Plg-R_KT_ (Plg-R_KT_-OEX) were lower than those of control mice (CagRosaPlgRKT). After 10 weeks on the HFD, CagRosaPlgRKT and Plg-R_KT_-OEX mice had similar body weights. However, Plg-R_KT_-OEX mice showed a more metabolically favourable body composition phenotype. Plg-R_KT_-OEX mice also showed improved glucose tolerance and increased insulin sensitivity. We found that the improved metabolic functions of Plg-R_KT_-OEX mice were mechanistically associated with increased energy expenditure and activity, decreased proinflammatory adipose macrophages and decreased inflammation, elevated brown fat thermogenesis, and higher expression of adipose PPARγ and adiponectin. These findings suggest that Plg-R_KT_ signalling promotes healthy adipose function via multiple mechanisms to defend against obesity-associated adverse metabolic phenotypes.

## Introduction

The obesity epidemic is a global health crisis that has led to increased rates of metabolic diseases, including type two diabetes (T2D), hypertension, non-alcoholic fatty liver disease (NAFLD), and cardiovascular complications. Adipose tissue (AT) plays a vital role in maintaining metabolic homoeostasis, but obesity can lead to a dysfunctional AT that contributes to metabolic abnormalities at multiple levels. Obesity-mediated adipose dysfunction results from a combination of factors, including inflammation due to increased accumulation of AT macrophages (ATM) and other immune cells, adipose fibrosis, and adipocyte hypertrophy due to impaired adipogenesis [1–9]. These changes lead to several metabolic abnormalities, including local adipocyte insulin resistance, ectopic lipid accumulation and ultimately to systemic insulin resistance, and glucose intolerance, abnormalities that can eventually cause T2D [4,10–13].

Plg-R_KT_ is a structurally unique transmembrane plasminogen receptor, with a C-terminal lysine exposed on the cell surface that binds plasminogen, and enhances the activation of plasminogen to plasmin and localizes the proteolytic activity of plasmin on the cell surface [14]. This function is promoted by the physical association of Plg-R_KT_ with the urokinase plasminogen activator receptor (u-PAR) [14]. While plasmin is primarily responsible for the degradation of fibrin, it also regulates tissue/extracellular matrix (ECM) remodelling and cell motility, activation of metalloproteinases (MMPs) and release of growth factors associated with ECM fibrosis and inflammation, processes that profoundly affect adipose function and systemic metabolism [15–18]. Therefore, via promotion of plasmin activity on metabolically active cells, including adipocytes, macrophages, hepatocytes, and muscle cells, Plg-R_KT_ is spatially located to affect modulation of the cell surface and its microenvironment and substantially regulate metabolic functions. Plg-R_KT_ regulates macrophage polarization and cytokine release [19,20]. In genome-wide association studies, the human PLGRKT (C9orf46) gene was identified as a novel genetic locus for the pathophysiology of childhood obesity [21] and has also been found to be associated with polycystic ovarian syndrome [22], which in turn is associated with obesity, insulin resistance, and T2D [23–25].

In our previous study, we showed that Plg-R_KT_ is highly expressed in both human and mouse AT. We also showed that HFD-fed Plg-R_KT_^−/−^ mice gained more body weight, were more insulin resistant/glucose intolerant, and developed more hepatic steatosis than HFD-fed Plg-R_KT_^+/+^ littermates [26]. These metabolic abnormalities were mechanistically associated with decreased adipogenesis, increased adipose inflammation, adipose macrophage accumulation, and fibrosis. In this study, we investigated whether overexpression of Plg-R_KT_ protects against HFD-induced adipose dysfunction and dysregulation of glucose homoeostasis.

## Methods

Mice studies were approved by Institutional Animal Care and Use Committees (The Scripps Research Institute and University of California, San Diego). To create mice overexpressing Plg-R_KT,_ we used Rapid-ROSA26 targeting to generate mice with the CAG Promoter Rosa26 PlgRKT knockin transgene (CagRosaPlgRKT) (Supplementary Fig.1(a-d)). The targeting vector contains a stop cassette that can be excised when crossed with Cre transgenic mice to produce overexpressing mice. We crossed CagRosaPlgRKT transgenic mice with B6.C-Tg(CMV-cre) mice (The Jackson Laboratory, Bar Harbor, ME) and bred the resulting F1 heterozygotes (Supplementary Fig. S1 (e – f)) to produce mice homozygous for the CagRosaPlgRKT transgene and expressing CMV-cre [CMV-cre X CagRosaPlgRKT mice (Plg-R_KT_-OEX)] to ubiquitously overexpress Plg-R_KT_.

All mice were in the C57Bl/6J background. Beginning at 8–10 weeks of age, male mice were placed on a HFD (D12492, 60% kcals from fat, Research Diets, New Brunswick NJ), and body weights were obtained weekly.

### Metabolic parameters

Mice were fasted for 6 h before performing the glucose tolerance tests (GTT), while insulin tolerance tests (ITT) were performed on non-fasted mice [27]. Glucose (2 g/kg BW) or human insulin (0.75 U/kg, Humulin; Eli Lilly, Indianapolis, IN) was injected i.p into mice, and blood was drawn via the tail vein at baseline and post-injection at indicated intervals. An insulin assay kit (Mercodia Ultrasensitive Insulin ELISA; Alpco Diagnostics, Salem NH) was used to measure plasma insulin levels, and glucose was monitored with the Easy Step Blood Glucose Diabetic Monitor Meter System. Total fat and lean mass were obtained via EchoMRI. Indirect calorimetry using a comprehensive laboratory animal monitoring system (CLAMS) was used to determine VO_2_, CO_2_, activity and food intake in individually housed mice [26,27]. Plasma alanine aminotransferase activity (ALT) and plasma leptin were determined by ELISA (Sigma).

### Insulin signalling analysis

Mice were injected with insulin (0.75 U/kg of human insulin) or saline via the tail vein and sacrificed 10 min post-injection. Tissues [liver, epididymal AT (EAT) and muscle] were collected, and analysed by western blot with antibodies to Akt (Cell Signaling Technology #9272), phospho-Akt (Cell Signaling Technology #9271, which detects endogenous levels of Akt only when phosphorylated at Ser 373), and β-actin (Cell Signaling Technology). UCP-1 protein expression in brown fat was determined by western blot analysis with antibodies to UCP-1 (Abcam, Waltham, MA). Tissues were lysed in RIPA buffer (Sigma) containing protease and phosphatase inhibitors (Thermo Fisher Scientific). Lysates were then electrophoresed and transferred to nitrocellulose membranes. The membranes were incubated with primary antibodies, washed with PBS-containing 0.1% Tween-20 and incubated with species specific IRDye®680RD/800CW-conjugated secondary antibodies. The Odyssey Imaging System (LI-COR) was used to visualize the immunoreactive bands, and densitometric quantification performed using Image Studio™ Lite Software 5.2 (LI-COR).

### Flow cytometry

EAT was minced in PBS with 0.5% BSA and incubated with collagenase (1 mg/ml in PBS/0.5% BSA) on a shaking platform at 37°C for 20 minutes. The mixture was filtered through a 250 µm filter, centrifuged for 5 min at 200 g, and the pellet recovered as the stromal vascular fraction (SVF) [26–29].

EAT-derived SVF cells were analysed by Flow cytometry as previously described [26–29]. Briefly, cells were stained at 4°C for 30 min with fluorophore-labelled monoclonal antibodies to F4/80, CD11c, and CD11b (eBioscience) in the presence of Fc receptor blocking antibodies (anti-CD16/CD32; eBioscience). After washing, cells were fixed in 1% formaldehyde and analysed on an LSR-II cytometer (BD Biosciences) and data processed using FlowJo.

### Real-time quantitative RT-PCR

Total RNA was isolated using the Ultraspec RNA isolation system (Biotecx Laboratories). cDNAs were synthesized from total RNA and analysed using gene-specific primer sets (Invitrogen) together with SYBR Green PCR Master mix (PerkinElmer) in an iCycler (Bio-Rad) [26–29]. Gene expression (relative levels) was calculated after normalization to β-actin using the ΔΔCT method (Bio-Rad).

Real-time quantitative RT-PCR primer sets used are as follows.

**PlgR**_**KT**_Left primer5’ ggc att gca acc atc tct tt 3’

Right primer5’ gtt ccg tag ccc agg tca ta 3’

**β-actin**Left primer5’ tgg aat cct gtg gca tcc atg aaa c 3’

Right primer5’ taa aac gca gct cag taa cag tcc g 3’

**TNFα**Left primer5’ cgt cag ccg att tgc tat ct 3’

Right primer5’ cgg act ccg caa agt cta ag 3’

**IL6**Left primer5’ gac aac cac ggc ctt ccc ta 3’

Right primer5’ gcc tcc gac ttg tga agt ggt 3’

**CCL2/MCP-1**Right primer5’ agc acc agc caa ctc tca c 3’

Left primer5’ tct gga ccc att cct tct tg 3’

**PPARγ** Left primer5’ ctg tcg gtt tca gaa gtg cct 3’

Right primer5’ ccc aaa cct gat ggc att gtg aga ca 3’

**Adiponectin**Left primer5’ ctc ctg ctt tgg tcc ctc ca 3’

Right primer5’ gtg cca tct ctg cca tca cg 3’

### Histology

Adipose tissue and liver were fixed in formalin and embedded in paraffin. Formalin-fixed, paraffin-embedded tissue sections were stained with H & E, and quantitative analysis of data was performed using Qupath v.0.3.2., an open-source software platform for image analysis.

### Statistical analysis

The statistical significance of differences between groups was assessed using repeated measures ANOVA and unpaired Student’s t-test. Repeated measures ANOVA was used to assess changes over time (example: body weight, GTT, ITT, etc.), while unpaired Student’s t-test was used to compare differences between groups at one point.

## Results

### Body composition and body fat distribution are improved in high fat diet (HFD)-fed mice overexpressing Plg-R_KT_ (Plg-R_KT_-OEX)

To create mice overexpressing Plg-R_KT,_ we used Rapid-ROSA26 targeting to generate mice with the CAG Promoter Rosa26 PlgRKT knockin transgene (CagRosaPlgRKT) (Supplementary Methods and Supplementary Fig. S1). The ROSA26 locus has become established as the preferred docking site for the ubiquitous expression of transgenes. The targeting vector contains a stop cassette that is excised when crossed with Cre transgenic mice to produce overexpressing mice. We crossed CagRosaPlgRKT transgenic mice with B6.C-Tg(CMV-cre) mice and bred the resulting F1 heterozygotes to produce mice homozygous for the CagRosaPlgRKT transgene and expressing CMV-cre [CMV-cre X CagRosaPlgRKT mice (Plg-RKT-OEX)]. Plg-R_KT_ gene expression and protein expression were increased fivefold and 3.2-fold, respectively, in adipose tissue of Plg-R_KT_-OEX mice compared to CagRosaPlgRKT mice (Supplementary Fig. S2). Subcellular fractionation showed Plg-R_KT_ localization in the plasma membrane (Supplementary Fig. S2D) .

**Figure 1.**
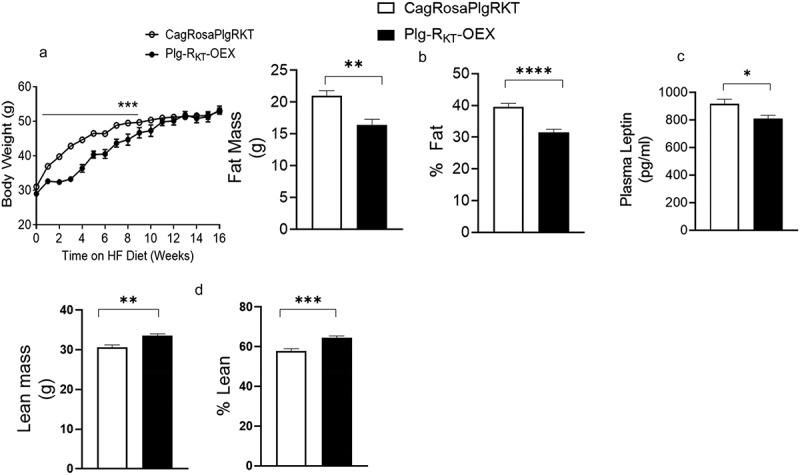
Plg-R_KT_ regulates body weight and adiposity. (a) body weights on the HFD of Plg-R_KT_-OEX and control CagRosaPlgRKT mice over the course of 16 weeks. (b) fat mass and % fat, (c) plasma leptin and (d) lean mass and % lean mass after 16 weeks on the HFD of Plg-R_KT_-OEX and CagRosaPlgRKT mice. For all panels, *N* = 8±SEM. *P < 0.05, **P < 0.01, ***P < 0.001, ****P < 0 0001.

Plg-R_KT_-OEX and control CagRosaPlgRKT mice were placed on a HFD and changes in body weight, fat, and lean mass were assessed. During 10 weeks on the HFD, the body weights of Plg-R_KT_-OEX mice were lower than CagRosaPlgRKT mice and this difference in body weight became distinguishable as early as 1 week on the HFD (Figure 1(a)). After 10 weeks, the weights of CagRosaPlgRKT and Plg-R_KT_-OEX mice were similar, and the final weights at 16 weeks were similar for both genotypes (Figure 1(a)). Echo MRI analysis of body composition measured after 16 weeks on the HFD revealed that Plg-R_KT_-OEX mice had a lower fat mass and per cent fat (Figure 1(b)) and increased lean mass and per cent lean mass (Figure 1(d)) than CagRosaPlgRKT controls. The decrease in fat mass was also reflected in the reduced levels of plasma leptin in Plg-R_KT_-OEX mice (Figure 1(c)).

**Figure 2.**
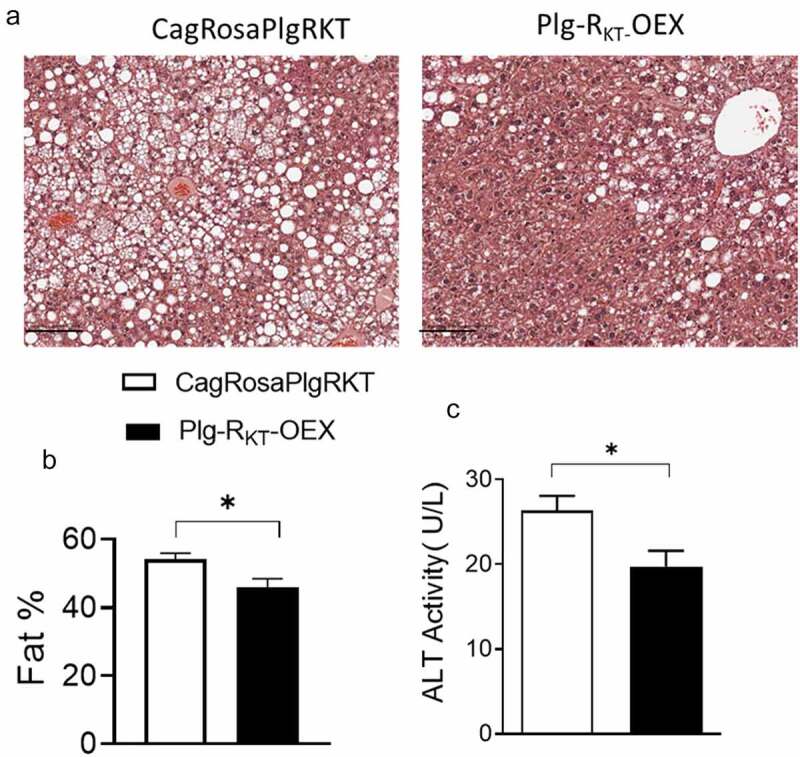
Plg-R_KT_ overexpression reduces HFD-induced hepatic steatosis. (a) Representative H &E-stained paraffin sections of liver from 16-week HFD-fed Plg-R_KT_-OEX and CagRosaPlgRKT mice. Scale bar is 100 μm (b) quantification of liver fat using QuPATH software and (c) plasma ALT activity of 16-week HFD-fed Plg-R_KT_-OEX and CagRosaPlgRKT mice. *N* = 8±SEM. *P < 0.05.

Obesity and adipose dysfunction in HFD-fed mice are often associated with ectopic lipid accumulation including hepatic steatosis [13,30]. H & E staining was performed on paraffin embedded sections of livers from HFD-fed CagRosaPlgRKT and Plg-R_KT_-OEX mice. Histological analysis showed that steatosis was significantly less in Plg-R_KT_-OEX mice compared with control CagRosaPlgRKT mice (Figure 2(a,b)). Likewise, decreased steatosis in HFD-fed Plg-R_KT_-OEX mice was associated with less hepatocellular damage, as indicated by a reduction in plasma ALT, an enzymatic marker of hepatocellular damage (Figure 2(c)).

**Figure 3.**
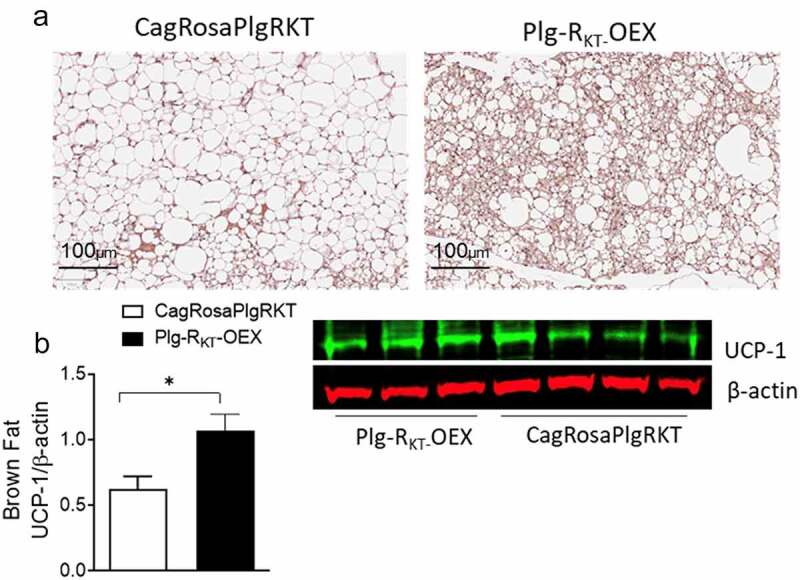
Plg-R_KT_ overexpression improves brown fat function. (a) Representative H & E-stained paraffin sections and (b) western blot of UCP-1 expression in brown fat of 16-week HFD-fed Plg-R_KT_-OEX and CagRosaPlgRKT mice. *N* = 4±SEM. *P < 0.05. Scale bar for panel a is 100 μm.

Brown adipose tissue (BAT) regulates systemic glucose homoeostasis and thermogenesis. In mice, feeding a HFD results in ‘whitening’ of the BAT, characterized by increased lipid deposition, reduced thermogenesis, and decreased expression of UCP-1. These changes typically lead to weight gain and dysfunctional glucose homoeostasis [31–34]. To investigate the effects of Plg-R_KT_ on BAT morphology and UCP-1 expression, H & E-stained sections of BAT were compared between HFD-fed CagRosaPlgRKT and Plg-R_KT_-OEX mice. BAT of HFD-fed control CagRosaPlgRKT mice showed adipocytes with a white fat-like morphology, with large unilocular lipid droplets (Figure 3(a)). In contrast, this ‘whitening’ phenotype was less in Plg-R_KT_-OEX, mice which displayed adipocytes containing multiple small lipid droplets, indicative of a more efficient brown fat depot (Figure 3(a)). In parallel with the change to a more brown fat-like morphology, UCP-1 expression was increased in BAT of HFD-fed Plg-R_KT_-OEX mice (Figure 3(b)).

Thus, despite the body weights at 16 weeks being similar in CagRosaPlgRKT and Plg-R_KT_-OEX mice, the overall body composition with an increase in lean mass, reduced steatosis and increased BAT activity reflected a metabolically healthier phenotype in HFD-fed Plg-R_KT_-OEX mice.

### Plg-R_KT_-OEX mice show enhanced overall energy expenditure after a HFD

**Figure 4.**
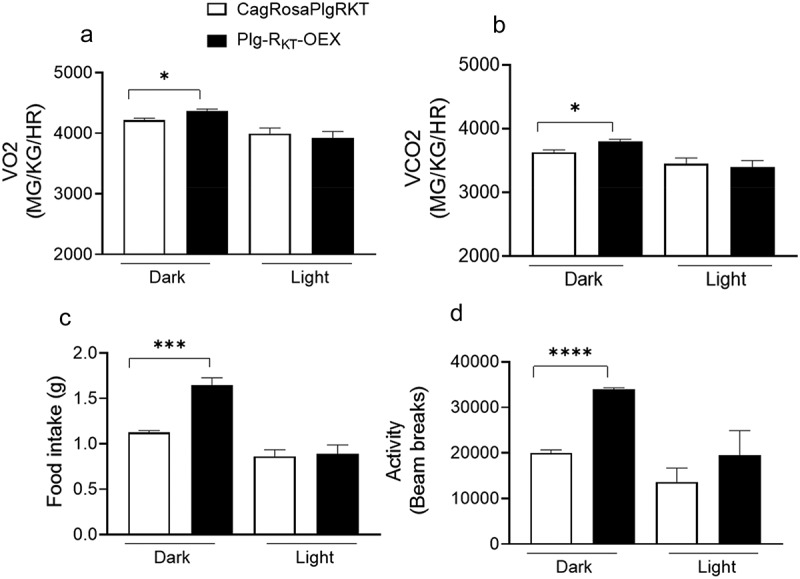
Plg-R_KT_ overexpression increases metabolism, food intake and activity. (a) O_2_ intake, (b) CO_2_ output, (c) food intake and (d) activity measured in the CLAMS of HFD-fed Plg-OEX and CagRosaPlgRKT mice. *N* = 5±SEM. *P < 0.05, **P < 0.001, ***P < 0 0001.

To compare energy expenditure, we measured metabolic parameters including O_2_ consumption, CO_2_ production, food intake and activity in HFD-fed CagRosaPlgRKT and Plg-R_KT_-OEX mice. Indirect calorimetry analysis using the comprehensive laboratory animal monitoring system (CLAMS) showed that O_2_ consumption and CO_2_ output were significantly greater in Plg-R_KT_-OEX mice only during the dark cycle, when the mice are active. No differences were observed between these genotypes for these parameters during the light cycle (Figure 4(a,b)). We also observed that the increased energy expenditure of Plg-R_KT_-OEX mice was associated with a higher food intake and activity during the dark cycle (Figure 4(c,d)).

### Adipose tissue inflammation is reduced in HFD-fed Plg-RKT-OEX mice

**Figure 5.**
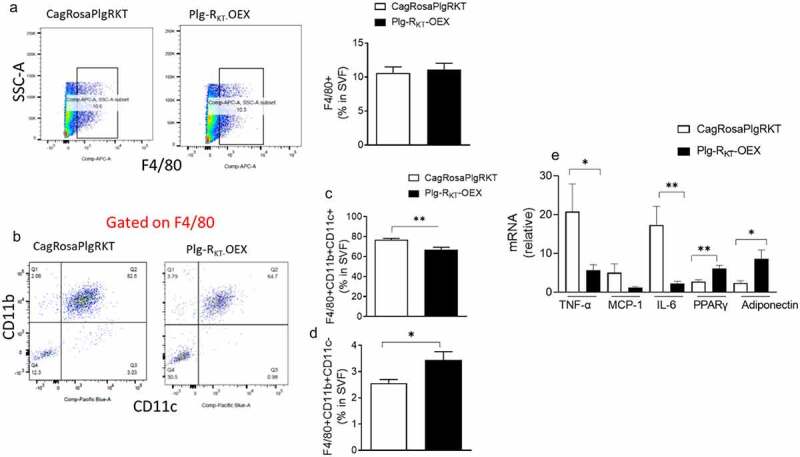
Overexpression of Plg-R_KT_ alters adipose macrophage polarization and reduces inflammation. (a) FACS quantification of F4/80+ cells and, (b, c, d) F4/80+/CD11b+/CD11c+ and F4/80+/CD11b+/CD11c- macrophage populations in the EAT of HFD-fed Plg-R_KT_-OEX and CagRosaPlgRKT mice. *N* = 6±SEM. (E) Cytokine/chemokine, PPARγ and adiponectin gene expression in EAT of HFD-fed Plg-R_KT_-OEX and CagRosaPlgRKT mice. *N* = 6±SEM. *P < 0.05, **P < 0.01.

We analysed the populations of macrophages in EAT of HFD-fed CagRosaPlgRKT and Plg-R_KT_-OEX mice. We found that the percentage of F4/80+ cells were the same in both groups (Figure 5(a)). However, the percentage of macrophages with the surface markers F4/80+/CD11b+/CD11c+ was significantly lower in EAT of HFD-fed Plg-R_KT_-OEX mice, while the percentage of macrophages with the surface markers F4/80+/CD11b+/CD11c- was significantly higher (Figure 5(a–c)). This shift in macrophage phenotype was associated with a decline in the proinflammatory cytokines TNF-α and IL-6 and the chemokine MCP-1 (Figure 5(e)). In addition, gene expression levels of the adipogenic marker PPARγ, and the insulin sensitizing molecule, adiponectin, were significantly higher in EAT of Plg-R_KT_-OEX mice than in control CagRosaPlgRKT mice (Figure 5(e)).

### Plg-R_KT_ overexpression improves glucose tolerance and insulin sensitivity

**Figure 6.**
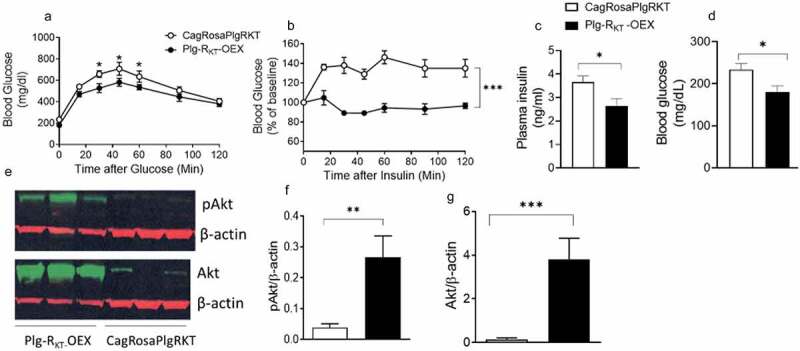
Overexpression of Plg-R_KT_ improves glucose tolerance and insulin sensitivity. (a) glucose tolerance test (b) insulin tolerance test (c) plasma insulin and (d) blood glucose of HFD-fed Plg-R_KT_-OEX and CagRosaPlgRKT mice. *N* = 8±SEM. (E,F,G) Representative western blots and densitometric scans of insulin-mediated levels of phosphorylated and non-phosphorylated Akt in EAT of HFD-fed Plg-R_KT_-OEX and CagRosaPlgRKT mice. *N* = 3–6±SEM. *P < 0.05, **P < 0.01, ***P < 0.001.

To test whether the metabolically favourable body composition, increased energy expenditure and decreased adipose inflammation in HFD-fed Plg-R_KT_-OEX mice mechanistically translated into improved glucose homoeostasis, we performed glucose tolerance tests (GTT) and insulin tolerance tests (ITT). In GTT, HFD-fed Plg-R_KT_-OEX mice were more efficient in clearing an intraperitoneal injection of glucose than control CagRosaPlgRKT mice (Figure 6(a)). In ITT, HFD-fed Plg-R_KT_-OEX mice also demonstrated increased insulin sensitivity, shown by more efficient insulin-mediated suppression of plasma glucose (Figure 6(b)). Plasma insulin and blood glucose levels were also lower in HFD-fed Plg-R_KT_-OEX mice than in CagRosaPlgRKT mice (Figure 6(c,d)) indicative of improved glucose homoeostasis.

To determine the tissue-specific contributions of Plg-R_KT_ overexpression to insulin signalling, we assessed the extent to which changes in the insulin signalling pathways in adipose, liver and muscle contributed to the overall increased systemic insulin sensitivity of HFD-fed Plg-R_KT_-OEX mice. In response to an insulin injection, the phosphorylation of Akt, a primary insulin signalling molecule, as well as the non-phosphorylated form of Akt was significantly greater in the EAT of HFD-fed Plg-R_KT_-OEX mice (Figure 6(e–g)). This was also observed in subcutaneous adipose tissue (Supplementary Fig. S3(a,b)) and muscle (Supplementary Fig. S3(c,d)) with both phosphorylated and non-phosphorylated Akt being significantly increased in response to insulin. In the liver, there was a trend towards an increase in phosphorylated and non-phosphorylated Akt after insulin injection (Supplementary Fig. S3(e,f)).

## Discussion

Previously, we showed that the pro-fibrinolytic plasminogen receptor, Plg-R_KT,_ plays a key role in adipose function and metabolic homoeostasis [26]. We found that this receptor is expressed in the AT and that its global deletion in mice (Plg-R_KT_^−/−^) led to exacerbated obesity and dysregulated glucose homoeostasis in response to a HFD. This metabolic dysfunction was mechanistically associated with impaired adipogenesis, increased adipose macrophage infiltration/inflammation, increased adipose fibrosis, dysregulated brown adipose thermogenesis and increased hepatic steatosis. The current study was designed to investigate the effect of Plg-R_KT_ overexpression on HFD-induced adipose function and metabolic homoeostasis. We found that HFD-fed mice with Plg-R_KT_ overexpression (Plg-R_KT_-OEX) had a metabolically favourable body fat distribution phenotype, improved glucose tolerance and increased insulin sensitivity. This improved metabolic phenotype of HFD-fed Plg-R_KT_-OEX mice was mechanistically related to 1) higher energy expenditure/metabolism and activity; 2) Increased brown AT thermogenic function; 3) a decrease in the population of ATM expressing the surface markers F4/80+/CD11b+/CD11c+ and an increase in the population of macrophages expressing the surface markers F4/80+/CD11b+/CD11c- in EAT, together with less inflammation and 4) higher expression of adipose adiponectin and PPARγ. These findings suggest that Plg-R_KT_ overexpression may be a potential therapeutic target for the treatment of obesity and metabolic disorders.

While the body weights of Plg-R_KT_-OEX and control CagRosaPlgRKT mice were similar at the end of 16 weeks of HFD-feeding, there were significant differences in body fat distribution, energy expenditure, and glucose homoeostasis. HFD-fed Plg-R_KT_-OEX mice had a lower fat mass, a higher lean mass, and a reduced accumulation of fat in the liver (hepatic steatosis). They also had a reduced ‘whitening’ of the BAT. BAT is a thermogenic fat depot that catabolizes glucose and fatty acids to produce heat and regulates systemic glucose homoeostasis and metabolism [31–34]. Brown adipose ‘whitening’ and reduced thermogenic capacity are characteristics of obesity [35,36]. The reduced ‘whitening’ and increased expression of the thermogenic protein, UCP-1, in BAT of HFD-fed Plg-R_KT_-OEX mice compared to control CagRosaPlgRKT mice, suggests that Plg-R_KT_ plays a unique role in BAT thermogenic function. These changes were also linked with increased metabolism/energy expenditure, improved glucose tolerance and insulin sensitivity in HFD-fed Plg-R_KT_-OEX mice. In addition, considering that Plg-R_KT_ overexpression significantly increased the thermogenic capacity of BAT, it may also have the potential to induce activation of beige adipocytes in the subcutaneous fat.

Accumulation of AT macrophages (ATM) and chronic inflammation in AT is a key feature of adipose dysfunction in both obese mice and humans [2,7,37,38]. Inflammatory cytokines such as TNF-γ and IL-6 as well as the chemokine CCL2, produced by proinflammatory M1-like AT macrophages and hypertrophic adipocytes inhibit local insulin signalling pathways to cause adipose and systemic insulin resistance [39–45]. Chronic AT inflammation also contributes to inhibition of adipogenesis resulting in adipocyte hypertrophy and ectopic lipid accumulation in other insulin target tissues such as the liver and muscle [9,46–50]. Cumulatively, these changes cause systemic metabolic dysfunction resulting in glucose intolerance, insulin resistance and T2D. We observed that in EAT of HFD-fed Plg-R_KT_-OEX mice, the population of ATM expressing the surface markers F4/80+/CD11b+/CD11c+ was significantly lower, whereas the population of macrophages expressing the surface markers F4/80+/CD11b+/CD11c- was higher. This was associated with a decrease in the proinflammatory cytokines TNF-α and IL-6, as well as the chemokine CCL2. Although ATM macrophages exhibit heterogeneity and complexity with regard to surface markers, taken together, these data are consistent with a shift from proinflammatory M1-like to M2-like macrophages. These results are consistent with the previously demonstrated role for Plg-R_KT_ in promoting macrophage polarization [20]. These changes were also associated with augmented expression of adiponectin and PPARγ, factors that promote adipogenesis and insulin sensitivity.

The higher levels of PPARγ and adiponectin in Plg-R_KT_-OEX mice are consistent with our previous findings, which suggest that Plg-R_KT_ plays a unique role in regulating adipogenesis [26]. Plg-R_KT_ expression increased during adipogenesis of 3T3-L1 pre-adipocytes, similar to expression of other mediators that drive adipogenesis [51,52]. Treatment of 3T3-L1 preadipocytes during differentiation with an inhibitory antibody to Plg-R_KT_ decreases the expression of PPARγ and several downstream adipogenic target genes [26]. Moreover, adipogenesis is decreased in Plg-R_KT_ deficient mice [26]. A previous study also showed increased plasminogen binding to adipocytes [53] and adipogenesis is reduced in both plasminogen deficient and u-PAR deficient mice [54,55].

Phosphorylation and activation of the kinase, Akt, is an essential step in the insulin signalling pathway for insulin-mediated glucose uptake. In HFD-fed Plg-R_KT_-OEX mice Akt phosphorylation in response to an insulin injection was significantly increased in the AT and muscle, and a trend towards an increase was also observed in the liver. These results suggest a primary role for Plg-R_KT_ signalling in the AT and muscle and to a lesser extent the liver in enhancing insulin-induced glucose uptake and maintenance of systemic glucose homoeostasis. The mechanism(s) by which overexpression of Plg-R_KT_ augments Akt phosphorylation are likely linked to decreased adipose inflammation. However, it is also possible that these insulin-sensitizing effects are facilitated via direct Plg-R_KT_ signalling. Although Plg-R_KT_ has only a short 4-amino acid cytoplasmic domain [14], Plg-R_KT_ signalling could be mediated via association with other signalling molecules such as the GPI-linked receptor u-PAR that regulates intracellular signalling [56].

Obesity is a chronic condition in which multiple mechanism(s) simultaneously contribute to its adverse metabolic phenotypes. However, increasing studies suggest that adipose dysfunction is a primary mechanism that initiates dysregulation of glucose homoeostasis [4,10–13]. Adipose dysfunction may arise due to defects in obesity-mediated adipogenesis resulting in adipocyte hypertrophy which blunts ‘healthy’ adipose expansion, and results in recruitment of macrophages and release of proinflammatory cytokines with the potential to further decrease adipogenesis and increase ectopic lipid deposition in tissues such as the liver and muscle [4,10–13]. Previous studies show that the development of dysfunctional AT in obesity is also accelerated by procoagulant and anti-fibrinolytic pathways leading to recruitment and proliferation of macrophages in the AT with subsequent development of the sequelae of obesity [27,57–62]. In the current study, we show that overexpression of the pro-fibrinolytic Plg-R_KT_ protects against adipose dysfunction and dysregulation of glucose homoeostasis. The mechanism(s) are related to decreased proinflammatory adipose macrophages, decreased inflammation, enhanced energy expenditure and brown fat thermogenesis, and increased adipogenesis (i.e. PPARγ, adiponectin). Our findings suggest that Plg-R_KT_ signalling contributes to the maintenance of adipose function and metabolic homoeostasis via multiple key regulatory steps and that overexpression of Plg-R_KT_ substantially mitigates the adverse metabolic effects of a HFD.

## Supplementary Material

Supplemental Material

## Data Availability

The data that support the findings of this study are available from the corresponding authors [L.A.M., R.J.P. and F.S], upon reasonable request.
