# Supplementary material for: Overexpression of Plg-RKT protects against adipose dysfunction and dysregulation of glucose homeostasis in diet-induced obese mice
Source: Adipocyte. 2023 Sep 4;12(1):2252729. doi: 10.1080/21623945.2023.2252729 (PMC10481882; doi:10.1080/21623945.2023.2252729)

**Supplementary Methods**

**Generation of Plg-RKT overexpressing mice using CAG Promoter Rosa26 Lox-Stop-Lox Knock-In targeting**

The vector was designed such that the expression of Plgrkt cDNA is driven by the pCAGGS promoter in the ROSA26 locus and is also controlled by a stop cassette (Supplementary Fig. 1A). The knock-in sequence was cloned into the Mlu I site of the ROSA26-pCAG-stop backbone vector using a conventional cloning method. The stop cassette in the ROSA-stop backbone vector contains a floxed PGK/gb2neoPGKpolyA2XSV40pA sequence. The pCAGGS promoter is placed immediately upstream of the stop cassette. The knock-in sequence is followed by a BGHpA sequence. The targeting vector contains a short homology arm (SA) with a 1.08 kb ROSA26 genomic sequence upstream of the pCAGGS promoter and a 4.34 kb long homology arm (LA) downstream of the BGHpA sequence. The targeting vector was confirmed by restriction enzyme analysis and sequencing after each modification. The junction of the pCAGGS promoter/ROSA26 genomic sequence was confirmed by sequencing with ROSASQ1. Primer ROSASQ2 reads from the ROSA26 genomic sequence into the Plgrkt -BGHpA sequence. The entire knock-in sequence and its junction with the stop cassette were fully sequenced with primer PLGR SQ1.

Primers used for sequencing:

ROSASQ1: 5’- AGC ACT TGC TCT CCC AAA GTC -3’

ROSASQ2: 5’- TGC TTA CAT AGT CTA ACT CGC GAC-3’

PLGR SQ1: 5’- ACG AGA AAG GCT GGC TTC TTT CG -3’

Ten micrograms of the targeting vector were linearized with Xho I enzyme and then transfected by electroporation of C57BL/6 (B6) embryonic stem cells. After selection with G418 antibiotic, surviving clones were expanded for PCR analysis to identify recombinant ES clones. Screening primer ROSAA1 was designed upstream of the short homology arm (SA) outside the 5’ region used to generate the targeting construct. PCR reactions using ROSAA1 with the PCAGR primer amplify a 1.42 kb fragment (Supplementary Fig. 1B). Clones 233, 242, 253, 341, and 361 were identified as positive and selected for further expansion (Supplementary Fig. 1C). After expansion, clones were reconfirmed for short homology arm integration using primers ROSA A1 and PCAGR and retention of cDNA using primers ROSA N2 and ROSA SQ2 (Supplementary Fig. 1D).

*Primers for PCR Screening*

ROSA A1: 5’- AAG AAG AGG CTG TGC TTT GGG –3’

PCAGR: 5’- CCG TAA ATA CTC CAC CCA TTG ACG –3’

ROSA SQ1: 5’- AGC ACT TGC TCT CCC AAA GTC –3’

ROSA N2: 5’- ATC ATG TCT GGA TCC CCA TCA AGC –3’

ROSA SQ2: 5’- TGC TTA CAT AGT CTA ACT CGC GAC –3’

Targeted iTL IC1 (C57BL/6) embryonic stem cells were microinjected into Balb/c blastocysts. Resulting chimeras with a high percentage black coat color were mated to wild-type C57BL/6N mice to generate F1 heterozygous offspring. Tail DNA was analyzed by PCR and sequencing (as outlined in Supplementary Fig. 1E) from pups with black coat color. Heterozygous mice were identified with targeted integration (Supplementary Fig. 1F). F1 heterozygous mice were mated, and homozygous mice (CagRosaPlgRKT) were identified using real time PCR with specific probes designed for the gene (Transnetyx, Cordova, TN). We crossed CagRosaPlgRKT transgenic mice with B6.C-Tg (CMV-cre) mice (The Jackson Laboratory, Bar Harbor, ME) and bred the resulting F1 heterozygotes to produce mice homozygous for the CagRosaPlgRKT transgene and expressing CMV-cre [CMV-cre X CagRosaPlgRKT mice (Plg-RKT-OEX)].

Subcellular fractionation of 0.3 mg of adipose tissue to obtain cytosolic and plasma membrane fractions was performed by differential and density centrifugation using the Minute^TM^ Plasma Membrane Protein Isolation and Cell Fractionation Kit (Invent Biotechnologies) according to the manufacturer’s instructions.

**Supplementary Figure Legends**

**Supplementary Figure 1.** (A) Construction of a CAG Promoter Rosa26 Lox-Stop-Lox Knock-In targeting vector. (B) PCR strategy used to identify positive ES clones. (C) Clones 233, 242, 253, 341, and 361 were identified as positive and expanded and reconfirmed for SA integration. An “x” denotes expanded clones. DNA from an individual clone (before reconfirmation) was used as a positive control and denoted as a (+). No DNA was used as a negative control and denoted by a (--). Wild Type DNA was used as a negative control and denoted by a (wt). (D) reconfirmation of clones for short homology arm integration. (E) PCR strategy used to identify heterozygous mice. (F) The PCR product from ROSA N2/ ROSA SQ2 screening of F1 heterozygous mice was run on a 0.8% gel with a 1 kb ladder as reference. The expanded ES clone (242), which was used as a positive control, is denoted by a (+), and C57BL/6 WT DNA which was used as a negative control is denoted by a (-).

**Supplementary Figure 2.** (A) qPCR (N=5-6±SEM) and (B) western blot with anti-Plg-R_KT_ and anti-β-actin and (C) quantification (N=4±SEM) for Plg-R_KT_ expression in adipose tissue (EAT) from mice homozygous for the CAG Promoter Rosa26 Plg-R_KT_ knock-in transgene (CagRosaPlgRKT) and Plg-R_KT_-OEX mice. *P < 0.05. (D) western blot with anti-Plg-R_KT_ and anti-α-enolase of cytosolic (4μg) and plasma membrane (4μg) fractions of adipose tissue of Plg-R_KT_-OEX mice. The α-enolase marker was present in both cytoplasmic and plasma membrane fractions as previously described^14^.

**Supplementary Figure 3.** **Overexpression of** **Plg-R_KT_ improves insulin signaling in muscle and liver.**

Representative densitometric scans (from western blots) of insulin-mediated levels of phosphorylated and non-phosphorylated Akt in subcutaneous adipose tissue (A,B), muscle (C,D) and liver (E,F) of HFD-fed Plg-R_KT_-OEX and CagRosaPlgRKT mice. N=3-6±SEM. *P < 0.05.

**Supplementary Figure 1**

**
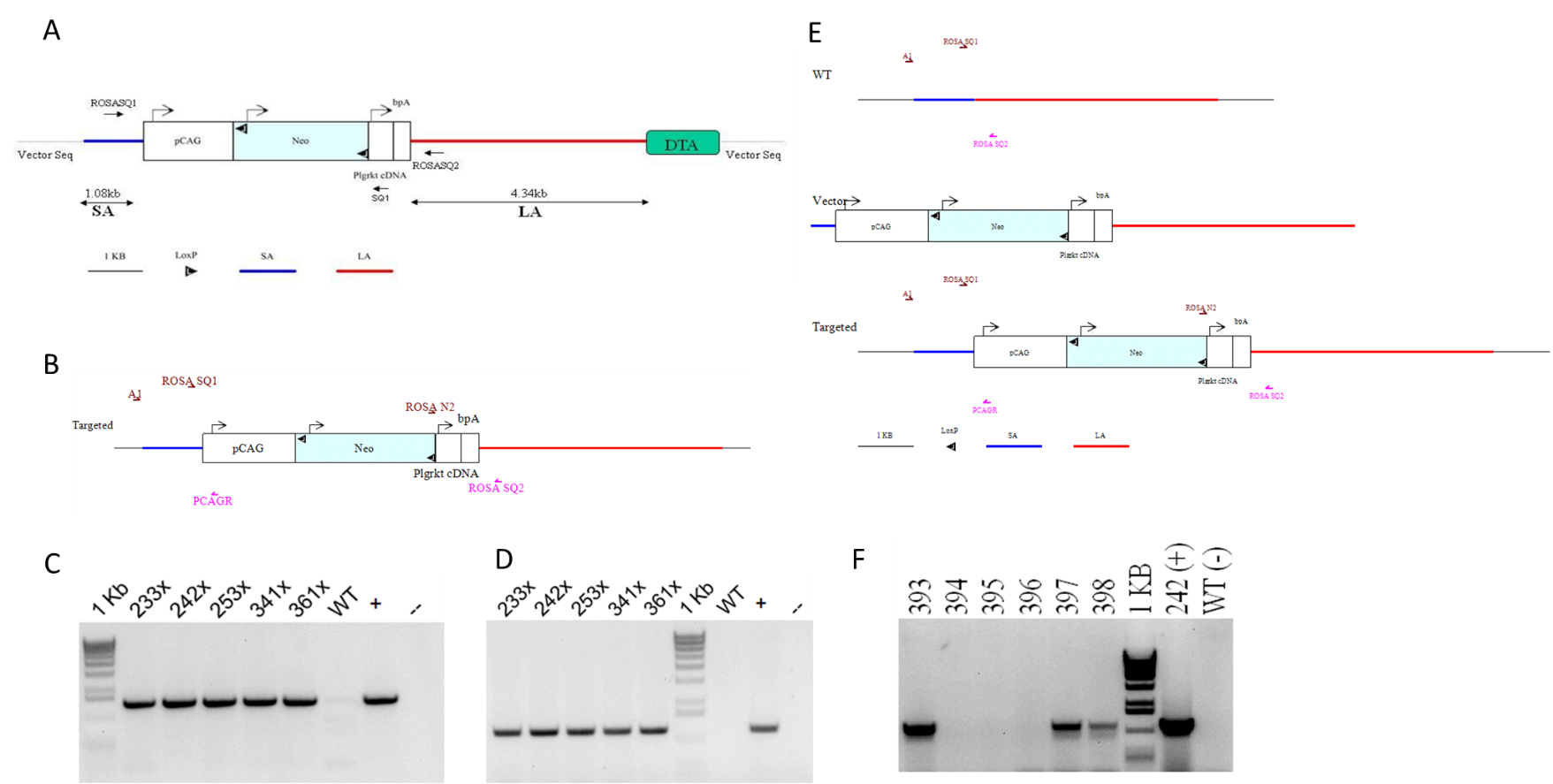
**

**Supplementary Figure 2**

**
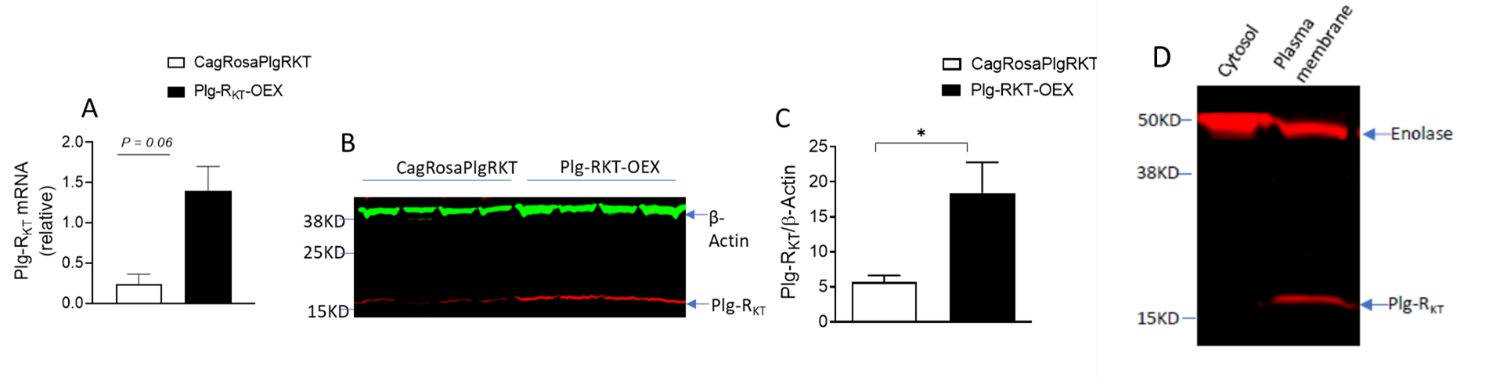
**

**Supplementary Figure 3**


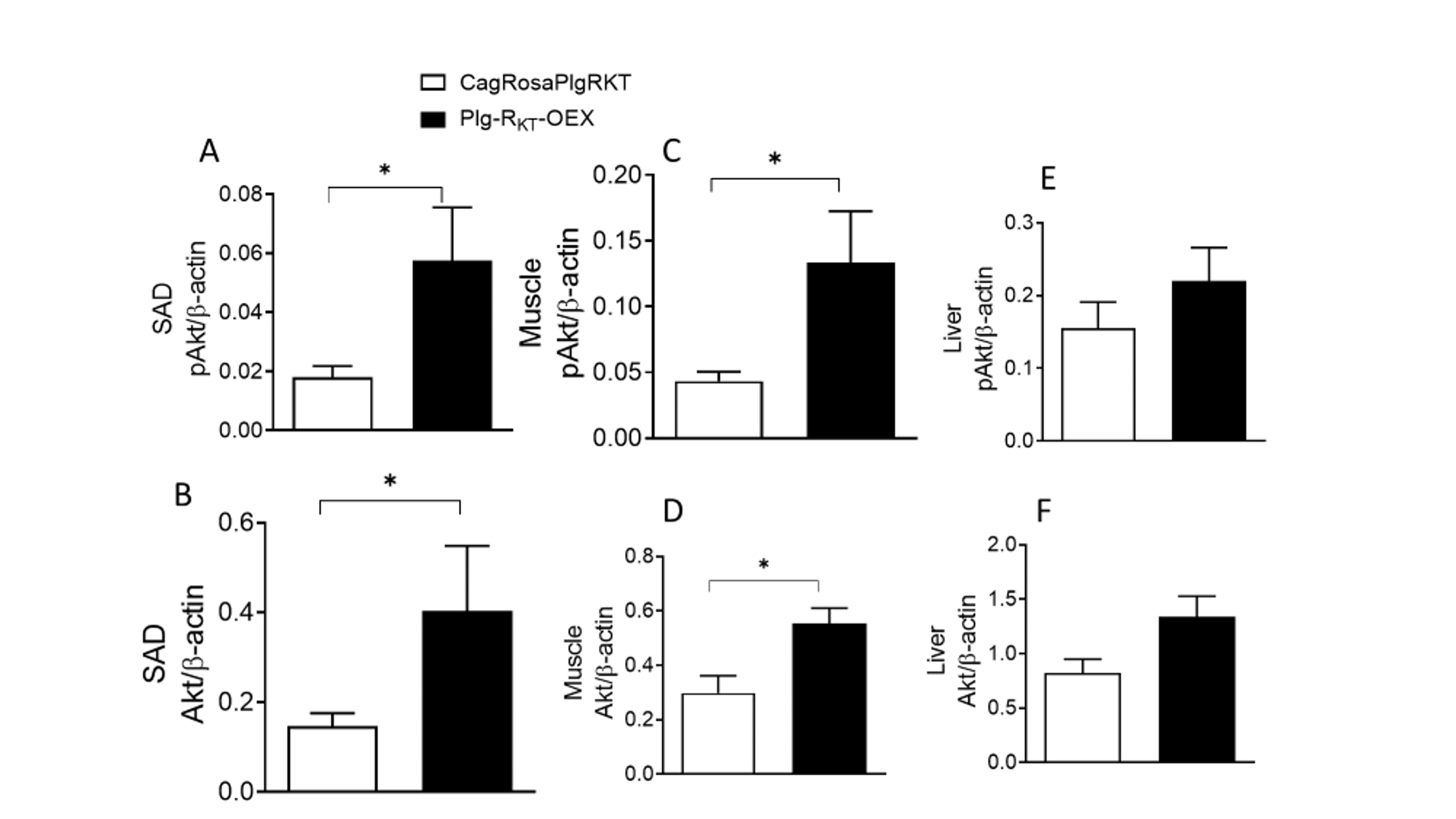

Supplement: Supplemental Material [file KADI_A_2252729_SM4436.docx]
